# Myricetin Suppresses the Propagation of Hepatocellular Carcinoma via Down-Regulating Expression of YAP

**DOI:** 10.3390/cells8040358

**Published:** 2019-04-17

**Authors:** Minjing Li, Jinliang Chen, Xiaofei Yu, Sen Xu, Defang Li, Qiusheng Zheng, Yancun Yin

**Affiliations:** 1 Institute of Integrated Medicine, Binzhou Medical University, Yantai 264003, China; liminjing512@126.com (M.L.); yxfbucm@126.com (X.Y.); lidefang@163.com (D.L.); 2 School of Basic Medical Sciences, Binzhou Medical University, Yantai 264003, China; chenjinliang0546@163.com; 3 Key Laboratory of Tumour Molecular Biology, Department of Clinical Medicine, Binzhou Medical University, Yantai 264003, China; xusenecho@163.com

**Keywords:** hepatocellular carcinoma, myricetin, LATS1/2-YAP, sensitization

## Abstract

Myricetin is a naturally occurring flavonoid with protective effects against a variety of cancers. However, the molecular mechanism of myricetin against hepatocellular carcinoma (HCC) has still not been fully elucidated. Previous studies have indicated that YAP is essential for cancer initiation and progression. However, whether YAP contributes to the anti-cancer effects of myricetin remains unclear. Herein, we aimed to investigate the effect of myricetin on HCC, and identify the underlying mechanisms. We report that myricetin induced apoptosis and proliferation inhibition in HepG2 and Huh-7 cells. Myricetin inhibited expression of YAP by promoting its phosphorylation and subsequent degradation. Myricetin inhibited YAP expression by stimulating kinase activation of LATS1/2. Knockdown expression of LATS1/2 by shRNA attenuated myricetin-induced phosphorylation and degradation of YAP. Furthermore, myricetin sensitized HCC cells to cisplatin treatment through inhibiting YAP and its target genes, both in vitro and in vivo. The identification of the LATS1/2-YAP pathway as a target of myricetin may help with the design of novel strategies for human HCC prevention and therapy.

## 1. Introduction

Hepatocellular carcinoma (HCC) is the fifth most frequent malignant cancer and the second leading cause of cancer-related death worldwide, according to the recently released cancer report [1]. Meanwhile, the overall number of deaths increased obviously due to HCC have rapidly increased in the past decades, especially in Western Europe and Northern America [1]. Most HCC patients presumably die within 9–12 months after diagnosis, due to resistance to routine chemotherapeutic agents, high recurrence, and metastasis [2,3]. Despite many attempts to exploit molecular targeted agents and immunotherapy methods, effective agents have not yet been developed for HCC treatment [4]. Therefore, effective therapeutic agents are urgently required for human HCC treatment.

There is increased interest in the use of natural compounds for the management of cancer and other diseases, based on their varied biological properties [5]. Myricetin (3,5,7-trihydroxy-2-(3,4,5-trihydroxyphenyl)-4-chromenone) is an edible flavonoid abundantly found in vegetables and fruits [6]. Studies have uncovered the different pathophysiological properties of myricetin, including its antioxidant, cytoprotective, antiviral, antimicrobial, and antiplatelet properties [6]. In addition, myricetin exhibits significant anticancer effects against various types of cancers, including ovarian cancer [7], breast cancer [8], gastric cancer [9], skin cancer [10], and placental choriocarcinoma [11]. Notably, it has been shown that myricetin induces cell-cycle arrest, proliferation inhibition, and apoptosis by targeting cyclin B/Cdc2 complex, Akt/p70S6K/Bad signaling, mitochondrial apoptotic pathways, and reactive oxygen species [12,13,14,15]. However, the underlying molecular mechanisms of these myricetin activities have yet to be clarified.

Yes-associated protein (YAP) and transcriptional co-activator with PDZ-binding motif (TAZ) are closely related transcriptional regulators pervasively expressed in human malignant tumors [16]. Studies indicate that YAP or TAZ is crucial for cancer initiation or propagation of the majority of solid cancers. The activation of YAP/TAZ leads to cancer stem cell attributes, growth, chemo-resistance, and metastasis [16]. Remarkably, up-regulated expression of YAP and TAZ highly correlates with poor tumor differentiation and unfavorable outcomes in HCC patients [17,18]. Moreover, overexpression of YAP in the liver in transgenic mice leads to formation of liver tumors [19]. Consistently, knockdown expression of YAP or TAZ significantly inhibits subcutaneous tumor growth of human and mouse HCC cell lines [18,20]. In this study, we identified whether YAP was a target of myricetin in human HCC cells. Moreover, we examined whether YAP was involved in the effect of myricetin against HCC. Herein, we show that myricetin decreased cell growth and increased apoptosis of HCC cells. Expression of YAP was down-regulated by myricetin treatment. In addition, myricetin promoted YAP degradation through the proteasome pathway, and thus inhibited expression of c-myc, survivin, and CYR61.

## 2. Materials and Methods

### 2.1. Reagents and Antibodies

The biochemical reagents myricetin (#SM8390) and cisplatin (#D8810) were purchased from Solarbio (Beijing, China). MG132 (#M8699) was purchased from Sigma (St. Louis, MO, USA). The plasmids pcDNA4/HisMaxB-YAP1 (#18978), pCIneoMyc-LATS1 (#66851), and pCIneoMyc-LATS2 (#66852) were purchased from Addgene (Watertown, MA, USA). The following antibodies were used: anti-Caspase 3 (66470-1-Ig), YAP (13584-1-AP), c-Myc (#10828-1-AP), survivin (#10508-1-AP), CYR61 (#26689-1-AP), LATS1 (#17049-1-AP), LATS2 (#20276-1-AP), CTGF (#23936-1-AP) and Actin (#60008-1-Ig) (Proteintech Group, Chicago, IL, USA); p-YAP-S127 (#13008) (Cell Signaling Technology, Beverly, MA, USA); and p-LATS1/2-S909/872 (#AF8163) (Affinity Biosciences, Cincinnati, OH, USA).

### 2.2. Cell Culture

HEK293T, normal hepatocyte cell LO2, the HCC cell line HepG2, and Huh-7 were obtained from the Cell Lines Bank, Chinese Academy of Science (Shanghai, China). The cells were maintained in DMEM containing 10% fetal bovine serum, 100 U/mL of penicillin, and 100 mg/mL streptomycin sulfate, and incubated at 37 °C in a humidified atmosphere of 5% CO_2_.

### 2.3. Cell Growth Assay

The cells were treated with myricetin or cisplatin, and then cell growth was tested. For cell counting assay, cells were seeded into 12-well plates (8000/well). Cell growth was evaluated by calculating the cell number with a TC20^TM^ automated cell counter (Bio-Rad, Hercules, CA, USA) at different time points. For the CCK-8 assay, HCC cells were seeded into 96-well plates at 5000 cells per well. The cells were treated with or without myricetin and cisplatin in five replicates. After 48 h, the cytotoxicity was assessed by incubating the cells with CCK-8 reagent (#CA1210, Solarbio) for 1 h, and measuring the absorbance at 450 nm with a microplate reader (Bio-Rad).

### 2.4. Cell Proliferation/Apoptosis Assay

The HCC cells were treated with or without myricetin and cisplatin, and then cell proliferation/apoptosis was tested. The EdU Kit (#C10310-1, Ribobio, Guangzhou, China) was used to test the proliferation of HCC cells, as we described previously [21]. In brief, cells were incubated with 50 μM EdU (#C10310-1) medium for 2 h followed by treatment with polyoxymethylene, glycine and TritonX-100. Then, the cells were incubated with Apollo^®^ 567 and Hoechst 33342. Images was gathered by fluorescence microscope (Olympus TH4-200, Tokyo, Japan). For the cell apoptosis assay, cells were collected after 48 h transfected. Then, the cells were stained by an Annexin V-PE/7-AAD apoptosis detection kit (#559783, BD Bioscience, San Jose, CA, USA), as we described previously [22]. Fluorescence signals from at least 10,000 cells were collected by FACS (Beckman, Fullerton, CA, USA) to determine the percentage of apoptotic cells.

### 2.5. Colony Formation Assay

Huh-7 cells were plated in 12-well plates at 1500 cells/well. The cells were treated with myricetin for 24 h, and then were allowed to grow in the absence of myricetin for 10 days to form colonies. The cells were fixed and stained with Giemsa dye liquor to visualize the cell colonies. Images were gathered by light microscope. The number and size of colonies was measured by a NIS-Elements F3.0 imaging system (Bio-Rad, Hercules, CA, USA). The relative number and size of colonies in six random visual fields (40×) under a light microscope is shown.

### 2.6. Quantitative Real-Time PCR

The cells were lysed, and total RNAs were obtained using Trizol Reagent (Invitrogen, Carlsbad, CA, USA). cDNAs were prepared with random hexamers from mRNA, using the AMV reverse-transcription kit (Promega Biotechnology, Madison, WI, USA). YAP and reference GAPDH were amplified by real-time PCR using the Power SYBR Green PCR master amplification mix (Life Technologies, Carlsbad, CA, USA) and 320 nmol/L primers. The primer sequences for human YAP were F: 5′-AGCTGCCCGACTCCTTCTTC-3′; R: 5′-GAGGAATGAGCTCGAACATGC-3′. The primer sequences for human GAPDH were F: 5′-AACTTTGGCATTGTGGAAGGA-3′; R: 5′-AACATCATCCCTGCTTCCAC-3′. Quantitative PCR reactions were performed on the iQ5TM (Bio-Rad, Hercules, CA, USA) for 35 cycles using a recommended annealing temperature (60 °C). Relative quantification with the comparative threshold cycle (Ct) was done using the Ct method.

### 2.7. Lentivirus Construction and Infection

The GFP tagged lentiviral vector PLL3.7 was used to express shRNAs designed to target LATS1/2. The LATS1 shRNA target sequence was: 5′-GAGAAATTAAGCCATCGTGTT-3′; 5′-CAAGTCAGAAATCCACCCAAA-3′; 5′-CACGGCAAGATAGCATGGATT-3′. The LATS2 shRNA target sequence was: 5′-CTACTCGCCATACGCCTTTAA-3′; 5′-CAGGACCAAACAGTGACACTT-3′; 5′-GCCATGAAGACCCTAAGGAAA-3′.

For lentiviral supernatant production, HEK-293T cells were grown on 10 cm culture plates to ~80% confluence. Then, 45 μL PolyJet reagent was diluted in 0.5 mL of DMEM medium (serum and antibiotic free), and incubated at room temperature for 5 min. In addition, 5.5 μg pMD2G, 2 μg PsPAX2, and 7.5 μg of the mixed shRNA construct were dissolved in 0.5 mL of DMEM medium (serum and antibiotic free), and mixed with the pre-diluted PolyJet. The mixture solution was incubated for 20 min before being added to HEK-293T cells. After 5 h, the supernatant was discarded and replaced with 10 mL of DMEM with 10% FBS. The supernatant, containing lentivirus, was harvested 48 h and 72 h later. After filtering, the lentivirus was used for infection. For infection, HCC cells were incubated with the LATS1/2 lentivirus supernatant for 6 h. The culture medium was discarded, and the cells were cultured in complete DMEM medium with 10% FBS for 24 h. This procedure was repeated for a second infection.

### 2.8. Western Blot

The cells were lysed with ice-cold RIPA lysis buffer containing protease inhibitors (PMSF, Aprotinin and phosSTOP). Lysate was incubated on ice for 30 min and then centrifuged for 20 min at 12,000× *g* to remove debris. Proteins were boiled in 1× loading buffer for 10 min, protein samples were resolved by SDS-PAGE, and proteins were transferred electrophoretically to a PVDF membrane (250 mA, 90 min). The membranes were incubated with primary antibodies overnight at 4 °C, and appropriate HRP-secondary antibodies for 1 h at room temperature. Detection was performed with chemiluminescent agents. Images were gathered by Alpha Innotech’s FluorChem imaging system. Densitometric analysis of blots was performed with Image J. All experiments were repeated three times with similar results. A representative experiment is shown. The full images of all blots with molecular markers are given in Figures S2–S6.

### 2.9. In Vitro Kinase Assays

The in vitro kinase assay was performed essentially as previously described [23]. Briefly, HEK293T cells were transfected with the pCIneoMyc-LATS1, and pCIneoMyc-LATS2 plasmid for 72 h respectively, then treated with or without 100 μM myricetin for 1 h. Then, cells were lysed and immunoprecipitated with Myc antibody. The immunoprecipitated recombinants myc-LATS1 and myc-LATS2 were used as kinase. HEK293T cells were transfected with the pcDNA4/HisMaxB-YAP1 plasmid for 72 h. Then, cells were lysed and immunoprecipitated with His antibody. The immunoprecipitated recombinant His-YAP was used as substrate. For immune-precipitation, the cells were harvested in the mild lysis buffer (MLB), respectively (10 mM Tris at pH 7.5, 100 mM NaCl, 10 mM EDTA, 0.5% NP-40, 50 mM NaF, 1.5 mM Na_3_VO_4_, protease inhibitor cocktail, 1 mM DTT and 1 mM PMSF). For each group, 1 mg of total proteins was immunoprecipitated with anti-His or anti-Myc antibodies for 90 min at 4 °C. Target proteins were collected by incubation with protein G Sepharose beads for 60 min at 4 °C. To remove low-affinity binding contaminants, the beads-proteins complexes were extensively washed three times with MLB, and once with kinase assay buffer (30 mM HEPES, 50 mM potassium acetate, and 5 mM MgCl_2_). Then, the immunoprecipitated his-YAP and immunoprecipitated myc-LATS1 or LATS2 were mixed, and incubated in a final volume of 90 μL at 37 °C in the kinase buffer, containing 500 μM ATP. Thirty minutes later, the reaction was stopped with the addition of 30 μL 4× loading buffer and boiled for 10 min. The phosphorylation of YAP was analyzed by Western blot.

### 2.10. Xenograft Tumor Growth Assay

All animal studies were performed according to the guidelines and approval of the Ethical Committee of Binzhou Medical University. To establish xenograft tumors, 5 × 10^6^ Huh-7 cells suspended in 100 μL phosphate-buffered saline were subcutaneously injected into BALB/c nude mice. At 10 days post-injection, the mice were randomly assigned into four groups. Then, myricetin (30 mg/kg/day), cisplatin (5 mg/kg/3 days) or both were intraperitoneally injected into the mice. The control group received an injection of vehicle. The size of the xenografted tumor was scaled per three days using a Vernier Caliper. When the tumor size of the control group reached approximately 10–15 mm, the mice were sacrificed, and the xenografts were resected. The xenograft tissues were subjected to TUNEL and Western blot analysis.

### 2.11. DNA Fragmentation Detection

Cell apoptosis in tumor tissues was analyzed by TUNEL assay using the Fluorescein-FragEL™ DNA Fragmentation Detection Kit (Calbiochem, San Diego, CA, USA) according to the manufacturer’s instruction. The nuclei of apoptotic cells were stained with highlight green fluorescence, and all cells showed blue fluorescence with DAPI. The apoptotic index was evaluated by the percentage of cells scored under a light microscope at 200-fold magnification.

### 2.12. Statistical Analysis

All data are presented as the means ± SD. A one-way ANOVA with the least significant difference post-hoc test was used to test for the differences in cell growth/proliferation/apoptosis, and colony survival rate. A two-way repeated measures ANOVA was used to test for the differences in tumor growth. A difference was defined as statistically significant if *p* < 0.05.

## 3. Results

### 3.1. Myricetin Suppressed Viability and Colony Formation of HCC Cells

**Figure 1:**
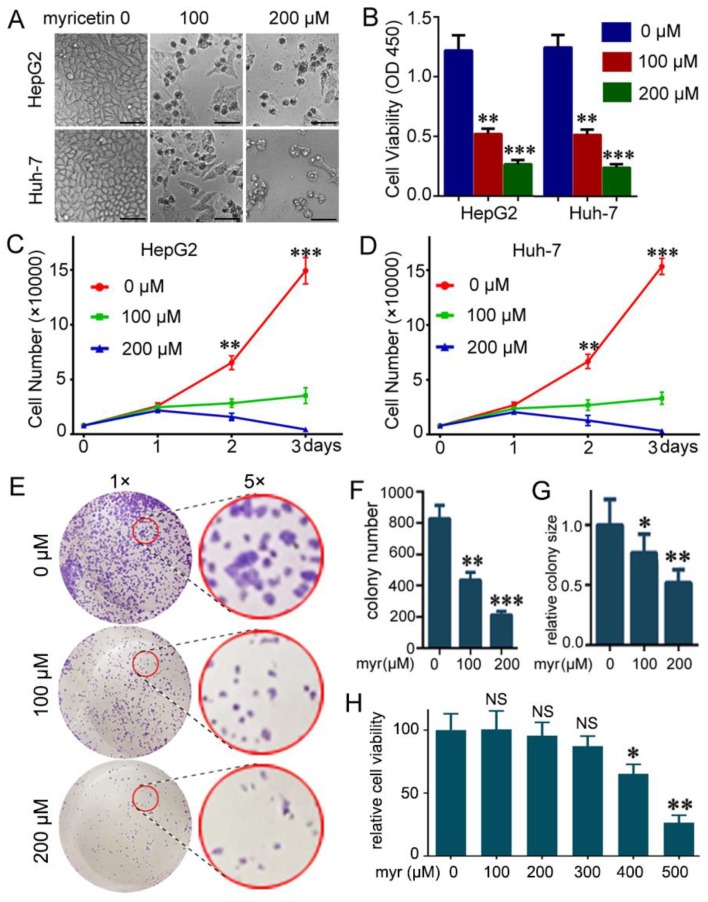
Myricetin suppresses viability of HCC cells. (**A**) Representative images of HCC cells at three days post-treatment with myricetin. Bar, 100 μm. (**B**) HCC cells were treated with the indicated dose of myricetin for 72 h. The cytotoxicity of myricetin was assessed by CCK-8. The absorbance at 450 nm (cell viability) is plotted. Values represent mean ± SD. *n* = 5, ** *p* < 0.005; *** *p* < 0.001, compared to control group. (**C**,**D**) Cell numbers at indicated days after treatment with myricetin. *n* = 5, ** *p* < 0.005, *** *p* < 0.001, compared to myricetin treated groups. (**E**–**G**) Huh-7 cells were seeded in 12-well plates (1500/well), and then treated with myricetin for 24 h after growing in the medium without myricetin for 10 days. Colonies were stained and quantified. (**E**) Representative images are shown. (**F**) colony number plotted. *n* = 3, ** *p* < 0.005, *** *p* < 0.001, compared to control group. (**G**) relative colony size was plotted. The colony size in the control group was set as 1. * *p* < 0.05, ** *p* < 0.005, compared to control group. (**H**) Normal hepatocyte LO2 cells were treated with the indicated dose of myricetin for 72 h. The cytotoxicity of myricetin was assessed by CCK-8. Relative cell viability was plotted. Values represent mean ± SD. *n* = 4; NS, not significant; * *p* < 0.05; ** *p* < 0.01, compared to control group.

To determine the potential cytotoxic effect of myricetin on HCC cells, CCK-8 assays were used to measure the growth viability in HepG2 and Huh-7 cells treated with different concentrations of myricetin for 72 h. As expected, myricetin treatment dramatically decreased cell growth and induced visible cell death in HCC cells (Figure 1A). Treatment of HepG2 and Huh-7 cells with 100 or 200 μM myricetin for 72 h markedly inhibited cell growth in a dose-dependent manner (Figure 1B). Furthermore, a cell counting assay showed that cell growth was much slower at two days post- myricetin treatment, and was more apparent at three days (Figure 1C,D). We next examined the effect of myricetin on colony formation ability of Huh-7 cells. There were fewer colonies formed in myricetin treated cells than in control cells (Figure 1E,F). Further, the colonies in myricetin treated cells tended to be smaller than those in control cells (Figure 1E,G). Overall, these results indicated the cytotoxic effects of myricetin on HCC cells. Moreover, we detected the effect of myricetin on LO2, a normal hepatocyte cell line. Little effect was detected on the viability of LO2 cells at concentrations of 50–300 µM for 72 h. Viability of LO2 cells was only dramatically decreased after incubation with 500 µM myricetin (Figure 1H). In addition, the IC_50_ values of myricetin in HepG2, Huh-7, and LO2 cell lines were listed in (Figure S1). Upon treatment with myricetin for 72 h, the mean IC_50_ values were 87.84 and 93.49 μM for HepG2 and Huh-7 cells, respectively. However, the IC_50_ for LO2 cells was almost five-fold of that for HepG2 and Huh-7 cells (Figure S1.)

### 3.2. Myricetin Induced Apoptosis and Reduced Cell Proliferation of HCC Cells

**Figure 2:**
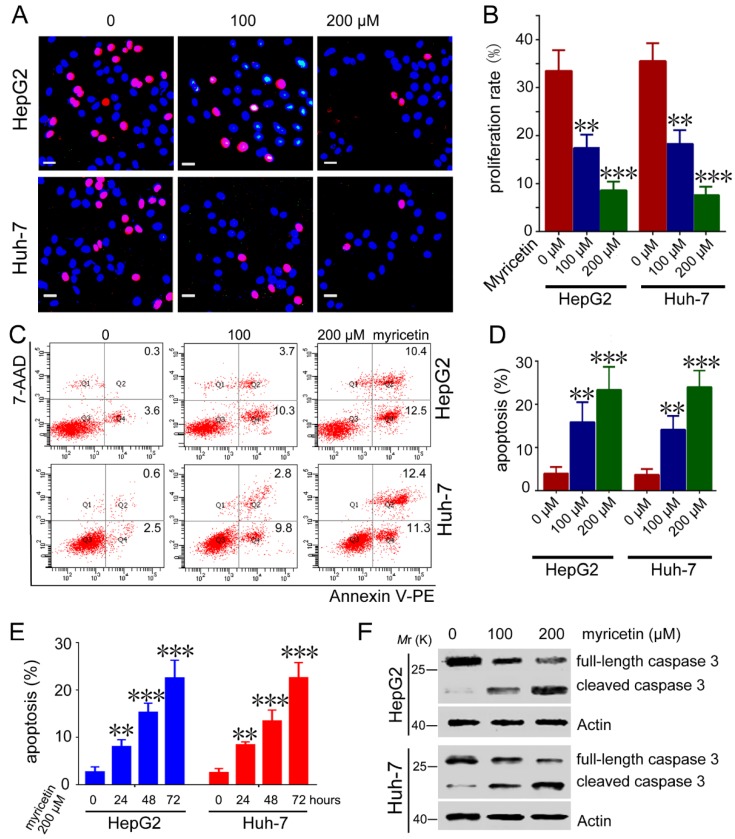
Myricetin induces proliferation inhibition and apoptosis in HCC cells. (**A**,**B**) Cells were treated with different doses of myricetin for 48 h, followed by EdU labeling analysis. Representative images are shown. EdU-labeled (red) indicates the proliferation of cells. Bar, 20 μm. The proliferation rate is plotted. *n* = 4, ** *p* < 0.005; *** *p* < 0.001, compared to control group. (**C**,**D**) Cells were treated with different doses of myricetin for 72 h, followed by flow cytometry analysis. Representative scatter plots are shown. The apoptosis percentage is plotted. *n* = 3, ** *p* < 0.005; *** *p* < 0.001, compared to control group. (**E**) HepG2 or Huh-7 cells were treated with 200 μM myricetin for 0, 24, 48, and 72 h, followed by flow cytometry analysis. The apoptosis percentage is plotted. *n* = 3, ** *p* < 0.005; *** *p* < 0.001, compared to control group. (**F**) HepG2 or Huh-7 cells were treated with myricetin for 72 h, followed by Western blot analysis of caspase 3 activation.

To detect the mechanisms underlying myricetin inhibiting cell growth, we compared the proliferation and apoptosis of HCC cells treated with myricetin. EdU-labeling assays showed that myricetin treatment dramatically inhibited proliferation of HepG2 and Huh-7 cells (Figure 2A,B). Furthermore, myricetin-treated HepG2 cells had a markedly increased apoptosis rate compared to control cells. Similar effects of myricetin on apoptosis were detected in Huh-7 cells (Figure 2C,D). Moreover, myricetin induced HepG2 and Huh-7 cell apoptosis in a time-dependent manner (Figure 2E). Conformably, cleaved caspase3 levels significantly increased in myricetin-treated HCC cells (Figure 2F). These results indicate that the dramatic slowdown of cell growth induced by myricetin treatment might be attributed to both apoptosis and proliferation.

### 3.3. Myricetin Decreased Expression of YAP and Its Target Genes

**Figure 3:**
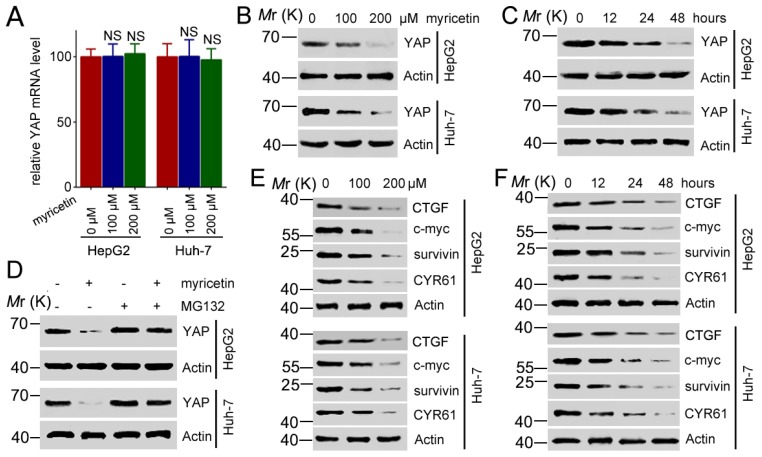
Myricetin stimulates degradation of YAP and inhibits CTGF, survivin, c-myc, and CYR61. (**A**) HCC cells were treated with the indicated dose of myricetin for 48 h, followed by RT-PCR analysis of *YAP* transcription. *YAP* mRNA levels were normalized to *GAPDH.* Values represent the mean ± SD. *n* = 3, NS, not significant, compared to control group. (**B**) Cells were treated with myricetin for 48 h at the indicated doses, followed by Western blot analysis of YAP expression. (**C**) Cells were treated with 200 μM myricetin for the indicated periods, followed by Western blot analysis of YAP expression. (**D**) Cells were pretreated with 10 μM MG132 for 5 h, then treated with or without 200 μM myricetin for 72 h, followed by Western blot analysis of YAP expression. (**E**) Cells were treated with myricetin for 48 h at the indicated doses, followed by Western blot analysis of indicated protein expressions. (**F**) Cells were treated with 200 μM myricetin for the indicated periods, followed by Western blot analysis of indicated protein expressions.

YAP/TAZ has been found to be involved in driving tumorigenesis and cancer propagation. To determine the molecular mechanism of myricetin-mediated anti-tumor activities, we measured the expression of YAP in mRNA, along with protein levels, in HCC cells treated with myricetin. The RT-PCR analysis demonstrated that *YAP* mRNA levels were invariant after myricetin treatment in HepG2 and Huh-7 cells (Figure 3A). However, treatment with myricetin stimulated YAP down-regulated expression at the protein level in a dose-dependent manner (Figure 3B). A time-dependent down-regulation of YAP by myricetin was also observed in HepG2 and Huh-7 cells (Figure 3C). These results indicate that myricetin might promote degradation of the YAP protein, since it had no effect on *YAP* transcription. To investigate this hypothesis, HCC cells were treated with myricetin and treated with or without MG132, a proteasome inhibitor, followed by Western blot analysis of the expression of YAP. Whereas myricetin treatment led to a decrease in the levels of YAP, treatment with MG132 blocked the down-regulation of YAP induced by myricetin (Figure 3D). These data suggest that myricetin promoted proteasome-mediated degradation of YAP. To further validate that the down-regulation of YAP by myricetin could impact on YAP downstream target genes, we detected the expression of CTGF (connective tissue growth factor), c-myc, surviving, and CYR61 in myricetin treated HCC cells. We found that myricetin downregulated expression of CTGF, c-myc, survivin, and CYR61 in both HepG2 and Huh-7 cells in a dose- and time-dependent manner (Figure 3E,F). Moreover, blocked YAP by shRNAs could decreased cell viability of HepG2 cells significantly, whereas blocked of YAP could not synergistically promotes myricetin-induced cell death (Figure S3). Taken together, these results suggest that myricetin might exert its anti-tumor activity through downregulation of the YAP signaling pathway.

### 3.4. Myricetin Promotes YAP Degradation by Stimulating LATS1/2 Activation

**Figure 4:**
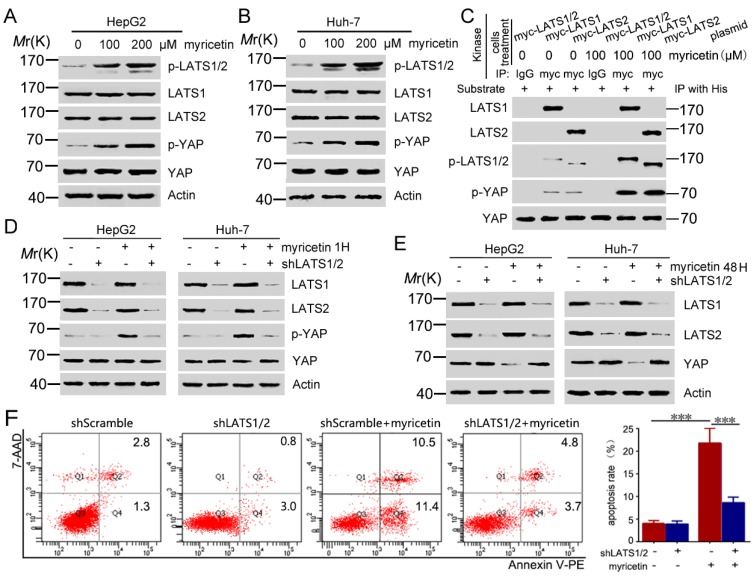
Myricetin promotes YAP phosphorylation and degradation via regulating LATS1/2 activity. (**A**,**B**) Cells were treated with myricetin for 1 h followed by Western blot analysis of the phosphorylation of the indicated proteins. (**C**) HEK-293T cells were transfected with the indicated plasmid for 72 h, and then treated with or without 100 μM of myricetin for 1 h, followed by immunoprecipitation with Myc or IgG (negative control) antibody, respectively. HEK-293T cells were transfected with the his-YAP plasmid for 72 h followed by immunoprecipitation with His antibody. The immunoprecipitated LATS1/2 proteins (kinase) were incubated with immunoprecipitated his-YAP (substrate) in a kinase assay system, followed by Western blot analysis. (**D**) HCC cells were transfected with LATS1/2 shRNA for 72 h, then treated with or without 200 μM myricetin for 1 h, followed by Western blot analysis of the phosphorylation of the indicated proteins. (**E**) HCC cells were transfected with LATS1/2 shRNA for 24 h, and then treated with or without 200 μM myricetin for 48 h, followed by Western blot analysis of the expression of the indicated proteins. (**F**) HepG2 cells were transfected with LATS1/2 shRNA for 48 h, and then were treated with 200 μM myricetin for 48 h, followed by flow cytometry analysis. Representative scatter plots are shown (left). The apoptosis percentage was plotted (right). *n* = 3, *** *p* < 0.001.

A previous study reported that phosphorylation of YAP by LATS1/2 promotes its proteasomal degradation [24]. To determine whether LATS1/2 is involved in myricetin-induced YAP degradation, HCC cells were treated with myricetin, followed by detection of transcription and expression of LATS1/2. Results show that myricetin did not affect the transcription (data not shown) and expression (Figure 4A,B) status of LATS1/2 at doses of up to 200 μM. However, treatment of HCC cells with myricetin resulted in an increase in LATS1/2 phosphorylation. Additionally, phosphorylation of YAP was increased (Figure 4A,B). We next determined whether myricetin regulates the kinase activity of LATS1/2 using an in vitro kinase assay. The LATS1/2 immunoprecipitated from myricetin-treated cells showed higher levels of serine phosphorylation and induced higher levels of YAP phosphorylation, compared to that from untreated cells (Figure 4C). This result demonstrates that treatment with myricetin led to an increase of the kinase activity of LATS1/2. Furthermore, knockdown expression of LATS1/2 by shRNA attenuated myricetin-induced phosphorylation of YAP (Figure 4D). Finally, silencing LATS1/2 by shRNA rescued myricetin induced down-regulated expression of YAP (Figure 4E). Conformably, the knockdown of LATS1/2 in HepG2 cells attenuated the cell apoptosis induced by myricetin (Figure 4F), further supporting our finding that LATS1/2 played important roles in myricetin induced degradation of YAP. In summary, our results indicate that myricetin regulated kinase activity of LATS/2, and thus stimulated YAP phosphorylation and degradation.

### 3.5. Down-Regulation of YAP by Myricetin Sensitized HCC Cells to Cisplatin

**Figure 5:**
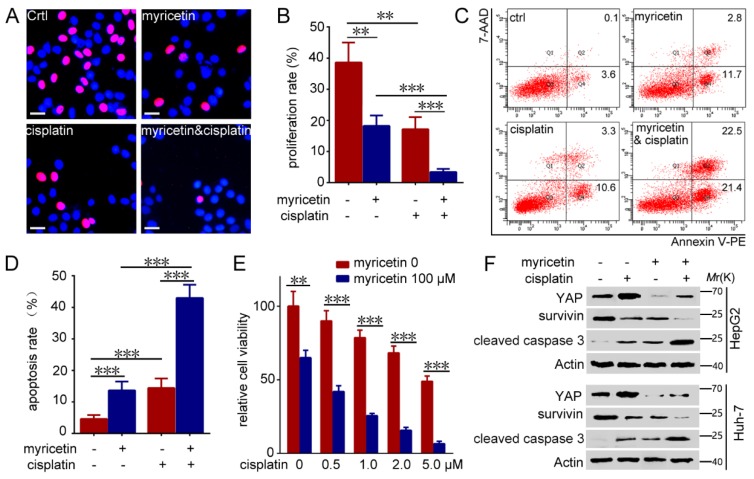
Myricetin sensitizes HCC cells to cisplatin by inducing degradation of YAP. (**A**–**D**) HCC cells were treated with or without 100 μM myricetin and 2 μM cisplatin for 48 h, followed by Hoechst and EdU labeling and flow cytometry analysis. (**A**) Representative images are shown. EdU-labeled (red) indicates proliferation of cells. Bar, 20 μΜ. (**B**) The proliferation rate is plotted. *n* = 4, ** *p* < 0.005; *** *p* < 0.001. (**C**) Representative scatter plots are shown. (**D**) The apoptosis percentage is plotted. *n* = 3, *** *p* < 0.001. (**E**) Huh-7 cells were treated with or without myricetin and cisplatin for 48 h. The cytotoxicity was assessed by CCK-8. The percentage of cell growth compared with control is plotted. Values represent the mean ± SD. *n* = 5, ** *p* < 0.005; *** *p* < 0.001. (**F**) HCC cells were treated with or without 100 μM myricetin and 2 μM cisplatin for 48 h, followed by Western blot analysis of the expression of the indicated proteins.

Cisplatin is commonly used as a chemotherapeutic agent for human HCC treatment. Chemo-resistance is a prime obstacle in cisplatin treatment of HCC. Consistently, recent studies have demonstrated that up-regulated expression of YAP confers resistance against cisplatin in oral squamous and urothelial cell carcinoma, and ovarian cancer [25]. The abovementioned results clearly demonstrate that myricetin could inhibited YAP activity. To determine whether myricetin could sensitize HCC cells to cisplatin, HepG2 cells were treated with myricetin, cisplatin or both, followed by testing of the cell proliferation and apoptosis. As expected, the results demonstrate that the combination of myricetin and cisplatin led to a significant decrease in cell proliferation (Figure 5A,B) and an increase in cell apoptosis (Figure 5C,D), compared to treatment with myricetin or cisplatin alone. Furthermore, the combination of myricetin and cisplatin can synergistically inhibit Huh7 cells growth (Figure 5E). Concordantly, treatment with cisplatin clearly induced up-regulated expression of YAP, whereas the combination of myricetin and cisplatin resulted in much lower levels of YAP than in cells treated with cisplatin alone (Figure 5F). Notably, the combination of myricetin and cisplatin resulted in much higher levels of cleaved caspase 3, and much lower levels of survivin, than in cells treated with cisplatin or myricetin alone (Figure 5F). Similar effects were observed in Huh-7 cells treated with myricetin and cisplatin (Figure 5F). Together, these results imply that YAP-induced cisplatin resistance could be reversed by myricetin in HCC cells.

### 3.6. The Combination of Myricetin and Cisplatin Synergistically Inhibits Tumor Growth In Vivo

**Figure 6:**
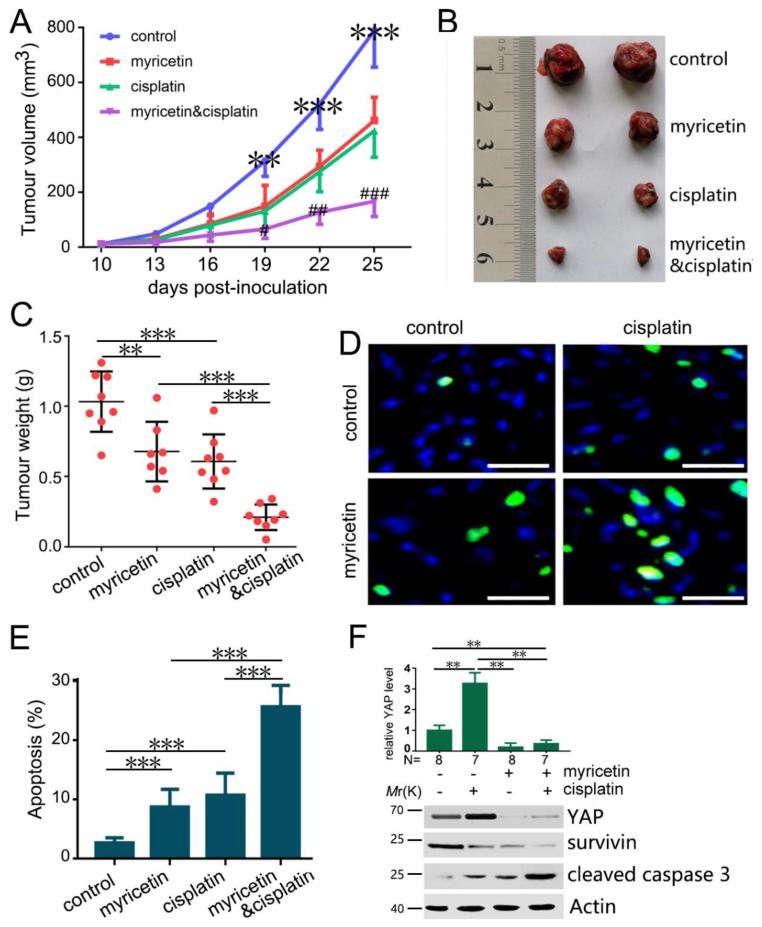
Myricetin sensitizes Huh-7 tumors to cisplatin in vivo. (**A**) Huh-7 cells were subcutaneously injected into BALB/C nude mice. The mice were treated with or without myricetin (30 mg/kg/day) and cisplatin (5 mg/kg/3 days). (*n* = 8 for control; *n* = 7 for myricetin group; *n* = 8 for cisplatin group; *n* = 8 for combination treatment). The effect of myricetin and cisplatin on Huh-7 tumor volume is plotted. ** *p* < 0.01, *** *p* < 0.005, compared to medication groups. # *p* < 0.05, ## *p* < 0.01, ### *p* < 0.005, compared to myricetin or cisplatin alone treatment groups. (**B**) Representative images of tumors (**C**) The effect of myricetin and cisplatin on Huh-7 tumor weight is plotted. ** *p* < 0.01; *** *p* < 0.005. (**D**) TUNEL analysis of apoptosis in Huh-7 tumors treated with or without myricetin and cisplatin. Bar, 20 µm. (**E**) The apoptosis rate is plotted. *** *p* < 0.005. (**F**) The levels of YAP, survivin, and cleaved caspase 3 proteins in tumors treated with vehicle, myricetin, and cisplatin were analyzed by Western blot. The relative content of YAP after normalization to actin is shown. Values represent the mean ± SD. ** *p* < 0.005.

To further evaluate the effects of myricetin and cisplatin in combination on tumor growth, we detected the tumor suppressive effect of myricetin and cisplatin in combination in Huh-7 xenografts in BALB/c nude mice. Whereas treatment with myricetin (30 mg/kg/day) or cisplatin (5 mg/kg/3 days) alone at the given dose moderately inhibited tumor growth, myricetin and cisplatin in combination suppressed tumor growth more obviously than myricetin or cisplatin alone (Figure 6A-C). Moreover, the apoptosis indices in the tumor tissue were detected by TUNEL assay. Consistent with in vitro studies, apoptosis of tumors treated with both myricetin and cisplatin was increased compared to that in tumors treated with myricetin and cisplatin alone (Figure 6D,E). Moreover, we examined the levels of YAP in these tumor xenografts. Cisplatin induced YAP expression, while myricetin suppressed cisplatin-induced YAP expression (Figure 6F). Moreover, the combination of myricetin and cisplatin resulted in much higher levels of cleaved caspase 3 and much lower levels of survivin than in tumors treated with cisplatin or myricetin alone (Figure 6F). These results demonstrate that myricetin significantly decreased cisplatin-induced YAP up-regulation, and enhanced the efficacy of cisplatin therapy in vivo. Therefore, the combination of myricetin and cisplatin may be a better choice for HCC treatment than cisplatin alone.

## 4. Discussion

**Figure 7:**
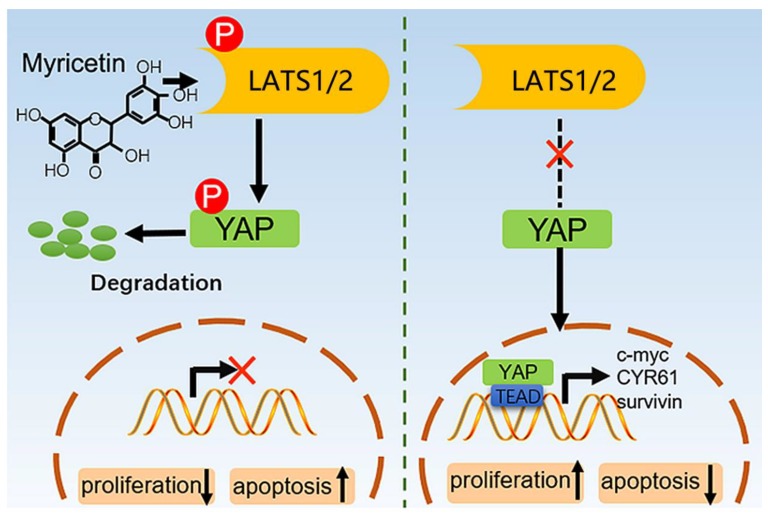
The model for myricetin regulating YAP expression. Myricetin stimulates phosphorylation and activation of LATS1/2 kinases, which in turn directly phosphorylate YAP on serine residues, leading to proteasomal degradation. Therefore, the transcription of the downstream effectors of YAP, such as CTGF, c-myc, CYR61 and survivin, are blocked.

Myricetin is a common plant-derived flavonoid, and is well recognized for its anti-cancer activities. Based on this study and other reports, myricetin acts against various types of cancers, including hepatic, skin, pancreatic, and colon cancer cells [6]. Myricetin displays its anti-cancer effects by altering some cancer hallmarks, including cell proliferation/apoptosis, angiogenesis, signaling pathways, and metastasis [26]. Notably, myricetin exerts its anti-cancer effects by directly interacting with oncoproteins such as AKT, MEK1, Fyn, and JAK1-STAT3 [10,27,28,29]. Our current study demonstrated that myricetin induced LATS1/2 activation, YAP phosphorylation and degradation, survivin inhibition, and caspase 3 cleavage, and subsequently induced proliferation inhibition and apoptosis of HCC cells (Figure 7). These findings uncover some novel molecular events in HCC cells after myricetin treatment.

The role of YAP as a novel promising therapeutic target has been pervasively recognized. Demonstrating the molecular mechanisms underlying YAP kinase activity regulation would have implications in the pathogenesis of human cancer. However, studies regarding specific YAP inhibitors or use of its potential therapeutic reagents in cancer treatment remain very limited. It has been reported that verteporfin combines with YAP and alters its conformation, thereby abrogating its interaction with TEAD2 [30]. Additionally, a peptide mimicking VGLL4 function has been detected to directly compete with YAP for binding TEADs in gastric cancer [31]. Notably, GCCSysm-4 has been discovered as a YAP and COX-2 dual inhibitor, efficiently suppressing both YAP and COX-2 activation in colorectal cancer [32]. Recently, Fisher, et al. found that sulforaphane increased YAP phosphorylation and proteolytic degradation in epidermal squamous cell carcinoma [33]. Despite these elegant observations, studies regarding YAP inhibitors for medicinal purposes remain very limited. Our current study firstly showed that myricetin significantly inhibited expression of YAP by promoting its phosphorylation and degradation. Meanwhile, it has been cumulatively demonstrated that specific inhibition of YAP is a promising and pharmacologically viable strategy in treating cancers with elevated expression of YAP [6,34]. Overall, this finding may help to shed some light on the development of new drugs, such as myricetin analogs that may inhibit HCC development by targeting YAP. Further study would be necessary to confirm whether myricetin inhibits expression of YAP in vivo, by using orthotopic xenograft models.

Previously, studies have demonstrated that the Hippo pathway component LATS1/2 is the key regulator of YAP. In general, upstream regulators such as cellular stress, mechanical signals, and extracellular stimuli promote activation of LATS1/2, which in turn phosphorylates YAP on multiple serine residues, resulting in either the degradation of YAP through the ubiquitin–proteasome pathway [24] or cytoplasmic retention of YAP by the 14-3-3 interaction [35]. Our in vitro kinase assays suggested that myricetin remarkably promoted activation of LATS1/2, and that the promotion leads to phosphorylation of YAP. Furthermore, shRNA against LATS1/2 rescued myricetin induced up-regulated phosphorylation, and down-regulated expression of YAP. These results indicate that myricetin-induced down-regulated expression of YAP depends on the kinase activity of LATS1/2. Remarkably, myricetin inhibits PAK1 expression via abrogating the Ras-mediated signaling [36]. Moreover, PAK1 antagonizes the phosphorylation and activation of LATS1/2, then activates YAP/TAZ mediated gene transcription via inducing inhibitory Merlin phosphorylation [37]. Consistently, these findings suggest that PAK1-LATS1/2 axis maybe involved in myricetin induced YAP/TAZ inhibition. More importantly, several reports have shown that LATS kinase activity is often constitutively decreased in cancer cells, and associated with increased cell proliferation [26]. It may be desirable to determine the effects of myricetin on LATS kinase activity for human HCC treatment. However, the phosphorylation and activation of LATS is context- and cell type-dependent [38]. Therefore, it would be necessary to define the different roles of myricetin in different types of cells and cancers.

Recent studies have suggested that LATS1/2 and YAP, as the key components of the Hippo pathway, may be closely related to the response of cancer cells to chemo-therapeutic drugs such as cisplatin, Taxol and sorafenib [16,39,40,41,42]. A promising pharmacological strategy for overcoming LATS1/2 and YAP induced chemotherapeutic resistance is urgently needed. Herein, we firstly showed that myricetin is a novel pharmacological inhibitor for LATS1/2 and YAP. We further discovered that myricetin significantly reawakened sensitivity of HCC to cisplatin treatment, both in vitro and in vivo. Conformably, Guo et al showed that the expression of YAP regulates sensitivity to sorafenib of HCC cells. Blocking of YAP by shRNAs synergistically promotes sorafenib-induced cell death in vivo [42]. These findings demonstrate that myricetin might be a potential option to combat chemo-resistance induced by aberrant activation of LATS1/2 and YAP. Striking, myricetin is affirmed as “GRAS” (generally recognized as safe) by the U.S. Flavor and Extract Manufacturer Association. Moreover, the no-observed-adverse-effect-level of myricetin is about 3000 mg/kg/day in rats [43]. The concentration of 30 mg/kg/day of myricetin might effectively inhibit cancer growth yet with minimal toxicity to normal cells. However, future studies are needed to define the prevalence of myricetin effects on YAP activation to understand the translation potential of myricetin treatments into the clinic.

## 5. Conclusions

Our study demonstrated that the inhibition of cell growth, induction of cell apoptosis, and proliferation inhibition in HCC cells by myricetin could be partly through the down-regulation of YAP expression. Myricetin stimulated phosphorylation and activation of LATS1/2 kinase, which in turn directly phosphorylated YAP on serine residues, leading to proteasomal degradation (Figure 6F). Furthermore, myricetin significantly sensitized HCC cells to cisplatin by inhibiting YAP and its downstream target genes. Overall, our findings suggest that inhibiting YAP by myricetin could be a potential effective approach to treating HCC, and overcoming chemoresistance.

## Supplementary Materials

The following are available online at https://www.mdpi.com/2073-4409/8/4/358/s1.
