# Supplementary material for: Myricetin Suppresses the Propagation of Hepatocellular Carcinoma via Down-Regulating Expression of YAP
Source: Cells. 2019 Apr 17;8(4):358. doi: 10.3390/cells8040358 (PMC6523269; doi:10.3390/cells8040358)
Supplement: Supplementary file 1 [file cells-08-00358-s001.pdf]

Supplementary Figures

	cell lines		
	HepG2	Huh-7	LO2
IC_{50} Values (μM) for 72 hours	87.84±8.77	93.49±7.33	454.85±32.47

Figure S1. IC50 values of myricetin on HepG2, Huh-7, and LO2 cell lines

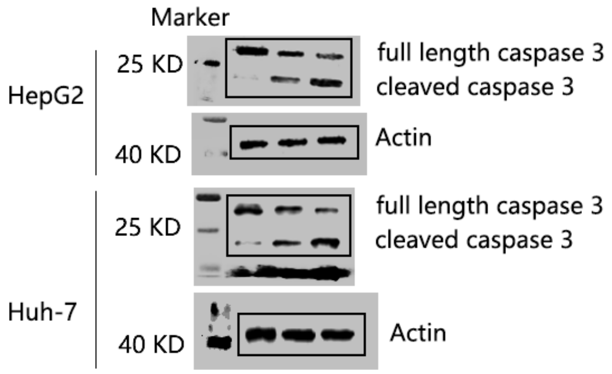

Figure S2. The full images of all blots with molecular markers for figure 2.

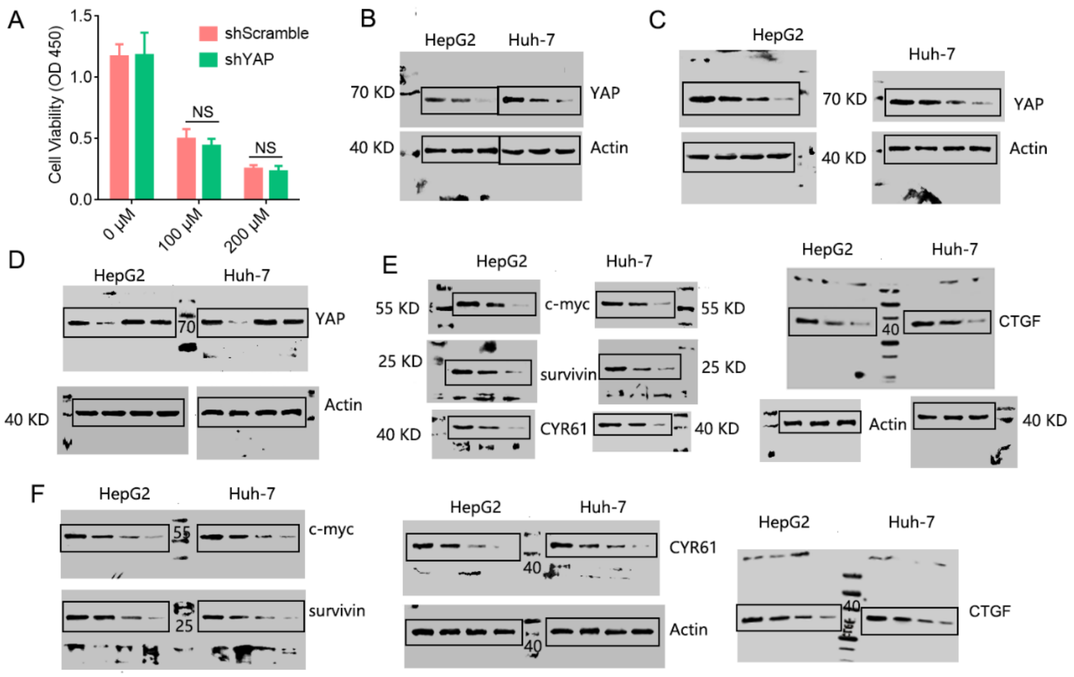

Figure S3. A, HepG2 cells were infected with YAP shRNAs, at 24 hours post-infection cells were treated with myricetin followed by CCK-8 analysis. B-F, The full images of all blots with molecular markers for figure 3.

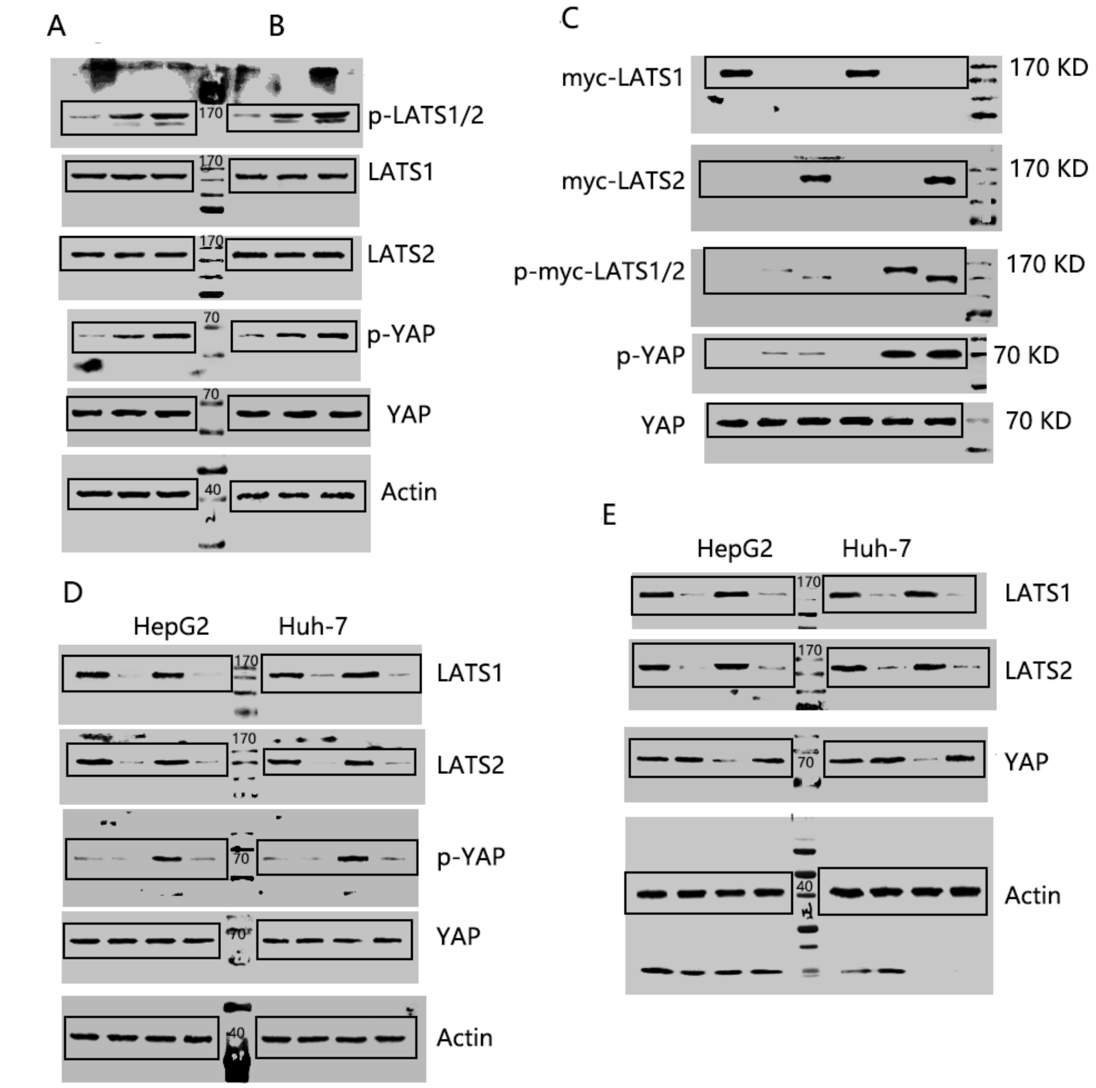

Figure S4. The full images of all blots with molecular markers for figure 4.

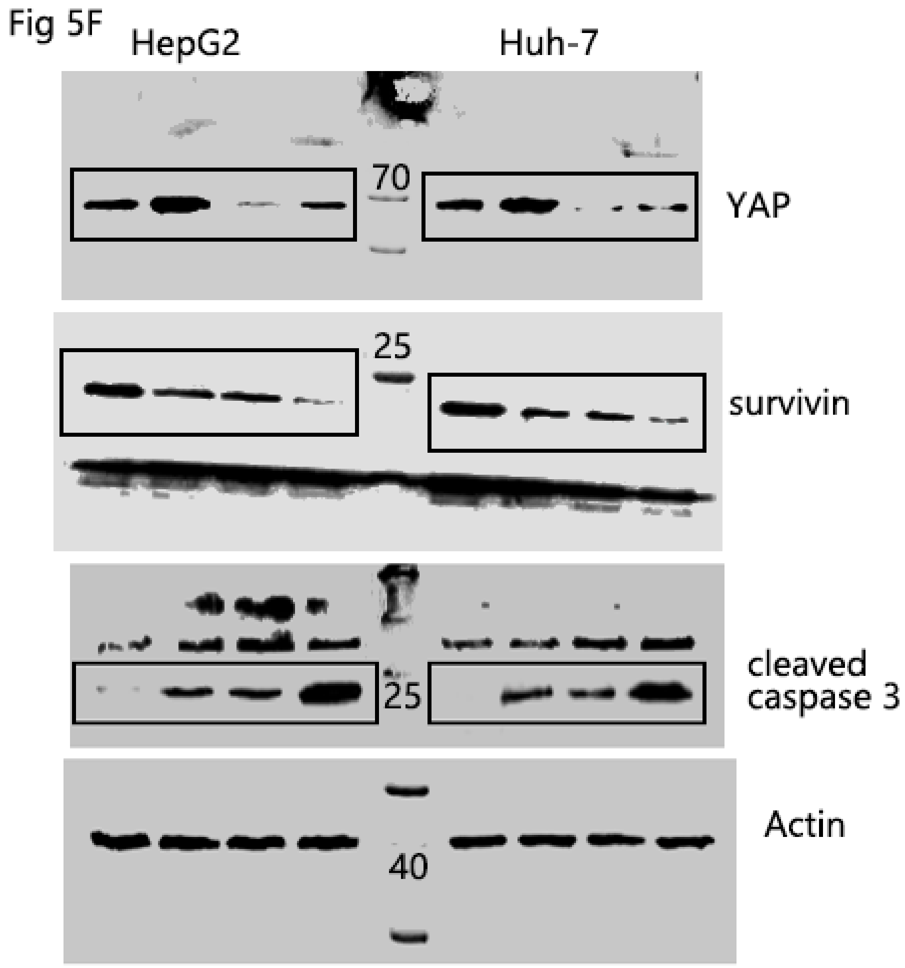

Figure S5. The full images of all blots with molecular markers for figure 5.

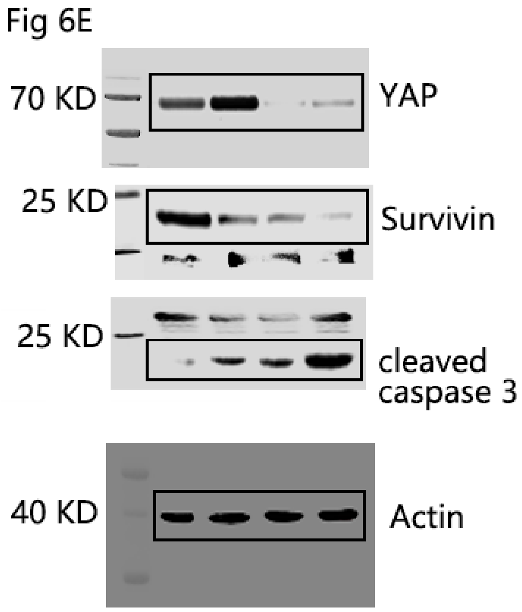

Figure S6. The full images of all blots with molecular markers for figure 6.
